# 

**Published:** 1891-07

**Authors:** 


### CONTENTS.

COMMUNICATIONS.

Preparation of Cavities and How to Fill Them........................... 317
Latest Theories of Dental Caries......... ...................................... 320
Preparing Sections of Teeth for Histology and Bacteriology........... 323
Hygiene of the Mouth.......................... .......................................... 335
Word-Blindness with Unusual Features.......................................... 340

PROCEEDINGS.

The Northern Ohio Dental Association.............................................. 341
An Anxious Doctor.......................................................................... 346

SELECTIONS.

Voice in Female Singers Affected by Gynæcological Disorders........ 347
The Centenary of the Microscope................................................... 348
Measuring the Perception of Oder.......................... ...................... 351
COMMENCEMENT EXERCISES.................................................. 352

EDITORIAL.

The American Dental Association................................................. 364
Cleveland, O., Dental Society........................... .......................... 366
Obituary........................................................................................ 367
In Memorium.................................................................................. 368

